# Potential complications when developing gene deletion clones in *Xylella fastidiosa*

**DOI:** 10.1186/s13104-015-1117-9

**Published:** 2015-04-16

**Authors:** Kameka L Johnson, Luciana Cursino, Dusit Athinuwat, Thomas J Burr, Patricia Mowery

**Affiliations:** Department of Plant Pathology and Plant-Microbe Biology, Cornell University New York State Agricultural Experiment Station, Geneva, NY 14456 USA; Department of Biology, Hobart and William Smith Colleges, Geneva, NY 14456 USA; Current address: Major of Organic Farming Management, Faculty of Science and Technology, Thammasat University, Pathum Thani, Thailand

**Keywords:** Aggregation, Pierce’s Disease, *Xylella fastidiosa*, Transformation, Antibiotic selection

## Abstract

**Background:**

The Gram-negative xylem-limited bacterium, *Xylella fastidiosa*, is an important plant pathogen that infects a number of high value crops. The Temecula 1 strain infects grapevines and induces Pierce′s disease, which causes symptoms such as scorching on leaves, cluster collapse, and eventual plant death. In order to understand the pathogenesis of *X. fastidiosa*, researchers routinely perform gene deletion studies and select mutants via antibiotic markers.

**Methods:**

Site-directed pilJ mutant of X. fastidiosa were generated and selected on antibiotic media. Mutant cultures were assessed by PCR to determine if they were composed of purely transformant cells or included mixtures of non-transformants cells. Then pure pilJ mutant and wildtype cells were mixed in PD2 medium and following incubation and exposure to kanamycin were assessed by PCR for presence of mutant and wildtype populations.

**Results:**

We have discovered that when creating clones of targeted mutants of *X. fastidiosa* Temecula 1 with selection on antibiotic plates, *X. fastidiosa* lacking the gene deletion often persist in association with targeted mutant cells. We believe this phenomenon is due to spontaneous antibiotic resistance and/or *X. fastidiosa* characteristically forming aggregates that can be comprised of transformed and non-transformed cells. A combined population was confirmed by PCR, which showed that targeted mutant clones were mixed with non-transformed cells. After repeated transfer and storage the non-transformed cells became the dominant clone present.

**Conclusions:**

We have discovered that special precautions are warranted when developing a targeted gene mutation in *X. fastidiosa* because colonies that arise following transformation and selection are often comprised of transformed and non-transformed cells. Following transfer and storage the cells can consist primarily of the non-transformed strain. As a result, careful monitoring of targeted mutant strains must be performed to avoid mixed populations and confounding results.

## Background

*Xylella fastidiosa* is a Gram-negative, xylem-limited, insect-vectored bacterium that is a causal agent of many economically important plant diseases, including Pierce’s disease of grapevines [1]. When infected vector insects probe plant tissues in search of the vascular xylem elements and sap contents, *X. fastidiosa* is subsequently transmitted to healthy plants. Once in the plant xylem, *X. fastidiosa* is postulated to migrate, attach, aggregate, and form biofilm that clogs the vessels leading to disease development.

*X. fastidiosa* migrate via twitching motility against the transpiration stream [2], which involves the extension and retraction of polar localized type IV pili [3]. *X. fastidiosa* cells are proposed to then attach to the xylem wall mainly using non fimbrial adesins, such as XadA (*Xanthomonas* adhesin-like protein A) and hemagglutinin proteins HxfB (hemagglutinin *Xylella fastidiosa* B) [4]. Cell-to-cell adhesion then occurs via non fimbrial adhesins HxfA, HxfB, XatA (*Xylella fastidiosa* autotransporter A) and the *X. fastidiosa* type I pili [4-7]. Type I pili co-reside at the cell pole with the long, fewer, type IV pili [8]. Finally biofilm formation commences [9].

To study the roles of *X. fastidiosa* genes and their encoded proteins, researchers traditionally delete *X. fastidiosa* genes using transposons or directed deletion with antibiotic-resistant markers [8,10]. These processes rely on identifying the mutant strain through antibiotic selection. *X. fastidiosa* Temecula 1 is sensitive to ampicillin, chloramphenicol, gentamicin, kanamycin, novobiocin, rifampin, and tetracycline [11]. Therefore plating transformants on these antibiotics should theoretically provide appropriate selectable markers for differentiation between wild-type *X. fastidiosa* and mutant strains. We recently discovered that non-transformed *X. fastidiosa* strains can survive on selectable medium, presumably due to spontaneous antibiotic-resistant mutants and/or extensive bacterial aggregation between transformed and non-transformed strains. As a result, strains presumed to be mutant clones are often a mixture of mutant and non-transformed *X. fastidiosa*, and over time, the non-transformed *X. fastidiosa* can become a significant population within a mixed sample.

## Methods

### Bacteria growth conditions

Wild-type *X. fastidiosa* Temecula 1 (kanamycin-susceptible) cells were grown on Periwinkle wilt (PW) agar [12] at 28°C for 7-10 days, in the absence of phenol red and with 3.5 g/L of bovine serum albumin (Invitrogen, Carlsbad, CA). *X. fastidiosa* mutants were grown on PW amended with kanamycin (50 μg/mL) (Sigma, St. Louis, MO). Cells were stored at -80°C in PD2 (Pierce’s Disease 2) media [13] with 7% DMSO (dimethyl sulfoxide).

### Construction of *X. fastidiosa pilJ* mutant

The *pilJ* mutant was constructed by double cross over recombination resulting in replacement of the *pilJ* gene with a kanamycin cassette as preciously described [10]. Approximately 500 bp (base pair) were amplified upstream and downstream of the *pilJ* gene using primers *pilJ*A/*pilJ*B and *pilJ*C/*pilJ*D respectively (Table 1). All primers were purchased from Sigma or Integrated DNA Technologies (Coralville, IA). The polymerase chair reaction (PCR) conditions were as follows: denaturation at 95°C for 2 min., 35 cycles of denaturation at 95°C for 30 sec., annealing at 55°C for 45 sec., and extension at 72°C for 1 min., followed by 72°C for 3 min. A 1-kb fragment was generated from the upstream and downstream fragment using splice extension overlap PCR using the conditions mentioned above with some modifications. Only the *pilJ*A/*pilJ*D primers were used with an annealing temperature of 63.5°C. The 1-kb fragment was cloned into pUC19 plasmid (Invitrogen) to generate pUC19-*pilJ*. A kanamycin cassette cloned from Topo vector pCR2.1 (Invitrogen) was excised from pGEM T-Easy (Promega, Madison, WI) using flanking the *Asc*I restriction sites. The pUC19-*pilJ* plasmid was digested with *Asc*I and the kanamycin cassette inserted into the 1 kb fragment. The presence of the *pilJ* deletion construct in pUC19 was confirmed by PCR. One microliter of the deletion construct was transformed into electro-competent *X. fastidiosa* [14]. Transformed cells were incubated in 1 mL PD2 broth for 24 hr before being plated onto PW agar plates amended with kanamycin (10 μg/mL) for 7-10 days. Target gene deletion was verified by PCR, using primers *pilJ*A/*pilJ*D or *pilJ*E/*pilJ*F, and the strain with *pilJ* gene deleted was designated Xf∆*pilJ*.

**Table 1:** **Oligonucleotide primers used in this study**

**Primer name**	**Primer sequence 5’ - 3’**	**Function**	**Reference**
*pilJ*A	ACCTGACTGTTCATCTGATGCG	Deletion of the *pilJ* gene and confirmation of deletion	This publication
*pilJ*B	TTCGGCGCGCCGAATCTAAATATGC	Deletion of the *pilJ* gene	This publication
	AAGACGGGACCG		
*pilJ*C	TTCGGCGCGCCGAAATGCTTCTCGG	Deletion of the *pilJ* gene	This publication
	CTTGGAAAGGA		
*pilJ*D	CGCAGCACGGATCTCGTTAA	Deletion of the *pilJ* gene and confirmation of deletion	This publication
*pilJ*E	CCCGAGTACCAACTTTTGGATTG	Amplification of *pilJ* gene fragment	This publication
*pilJ*F	ATCTGCTCATCCTTTCCAAGCC	Amplification of *pilJ* gene fragment	This publication
RST31	GCGTTAATTTTCGAAGTGATTCGAT TGC	*Xylella fastidiosa* detection	[17]
RST33	CACCATTCGTATCCCGGTG	*Xylella fastidiosa* detection	[17]

### PCR amplification of DNA to confirm deletion of *pilJ* gene

The PCR mix included 100 ng of DNA, 200 mM dNTP (deoxyribonucleotide triphosphates), 2 mM MgSO_4_, 0.5 U Platinum Taq (Invitrogen), and 40 nM each of primer (*pilJ*A/*pilJ*D or *pilJ*E/*pilJ*F) in a 25 μL reaction mixture. PCR conditions were as follows: denaturation at 95°C for 2 min., 35 cycles of denaturation at 95°C for 45 sec., annealing at 60°C for 15 sec., and extension at 72°C for 2 min. and 30 sec., followed by 72°C for 6 min. PCR fragments were separated by gel electrophoresis and visualized using the Bio-Rad GelDoc XR system (Bio-Rad, Hercules CA).

### Real-time (RT) PCR amplification to confirm deletion of *pilJ* gene

The real time PCR mix included 12.5 μL SybrGreen real-time PCR mix (Bio-Rad) and 40nM of each primer in a total of 25 μL. PCR conditions include denaturation at 95°C for 3 min., and 35 cycles of 95°C for 10 sec., 50°C for 5 sec., 72°C for 25 sec. The melt curve was calculated at 76-95°C with 0.5°C increments for 5 sec.

### Bacterial aggregation

Bacteria, *Escherichia coli* or *X. fastidiosa*, were grown on Luria Bertani (LB) or PW agar plates. Cells were removed from the plates and suspended in succinate-citrate-phosphate (SCP) buffer [15] to an OD_600_ of 0.10 (4×10^7^ CFU/mL). The cells were suspended vigorously by vortex mixer (Fisher Scientific, Springfield, NJ) for 5 minutes at maximum speed and by pipetting before being observed for aggregates. Cells were examined on a Axioskop 2 Plus microscope (Carl Zeiss Microscopy, Thornwood, NY) with a QImaging Retiga Ex*i* camera (QImaging, Surrey, Canada) at 40X using QCapture 2.9.13 software (QImaging).

### Mutant and wild-type *X. fastidiosa* on antibiotics

Wild-type *X. fastidiosa* and the Xf∆*pilJ* mutant cells were grown to an OD_600_ of 0.10 in PD2 liquid media. The Xf∆*pilJ* mutant was a pure mutant having undergone multiple rounds of isolation followed by RT-PCR confirmation of not containing mixed populations. Wild-type bacteria, the Xf∆*pilJ* mutant, or equal concentrations of both were suspended in PD2 media and incubated at 28°C for 24 hr, as occurs during a transformation [14]. After incubation, 100 μL was plated onto PW agar plates containing 0, 4, 10, 25, or 50 μg/mL kanamycin, and plates were incubated for 7-10 days at 28°C until growth was visible. Bacteria were scraped and collected from each plate and conventional PCR was conducted, as previously described. The PCR fragments obtained were analyzed by gel electrophoresis and visualized using the Bio-Rad GelDoc XR system (Bio-Rad). The experiment was performed three times.

## Results and discussion

**Figure 1:**
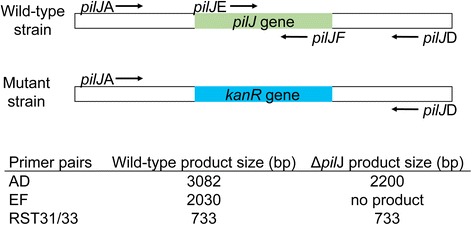
Orientation of primers for *Xylella fastidiosa pilJ* gene deletion. Location of binding sites for PCR primers and length of resulting PCR products for transformed XfΔ*pilJ* strains and for wild-type control or non-transformed cells. RST31/33 are primers specific to *X. fastidiosa* and used for bacteria confirmation.

**Figure 2:**
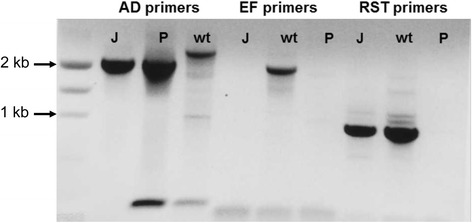
Confirmation of *Xylella fastidiosa pilJ* gene deletion. The *pilJ*A/*pilJ*D (AD) primers amplify a 3082 bp fragment from wild-type control cells (wt) or a 2200 bp fragment form the XfΔ*pilJ* mutant (J) and deletion plasmid (P). The *pilJ*E/*pilJ*F (EF) primers amplify a 2030 bp band for the wild-type control strain and no band for the mutant cells or deletion plasmid. RST31/33 (RST) primers confirm that the bacteria were *X. fastidiosa*.

The *pilJ* gene encodes a putative chemotaxis receptor of interest [16]. The gene was deleted from *X. fastidiosa* Temecula 1 using site directed replacement with a kanamycin resistant marker [10]. Transformants were selected on antibiotic plates at 10 μg/mL since the minimum inhibitory concentration of kanamycin for *X. fastidiosa* Temecula 1 is 4 μg/mL [11]. All subsequent work with the transformed cells (Xf∆*pilJ* mutants) were performed with 50 μg/mL kanamycin. Deletion of the *pilJ* gene was confirmed by PCR using multiple primer sets (Figure 1). The *pilJ*A/*pilJ*D (AD) primers amplified a 3082 bp band from wild-type control bacteria and a 2200 bp band from the deletion plasmid pUC19-*pilJ* and the Xf∆*pilJ* strain (Figure 2). The *pilJ*E/*pilJ*F (EF) primers are complementary to sequences within the *pilJ* gene producing a 2030 bp band for wild-type control cells and no fragments for the Xf∆*pilJ* bacteria or plasmid control. The *X. fastidiosa*-specific RST31/33 primers [17] confirmed that the bacteria were *X. fastidiosa.* As expected, these primers failed to amplify a band from the pUC19-*pilJ* plasmid. The Xf∆*pilJ* strain was subsequently tested in a number of behavioral assays to explore the role of the PilJ protein (data not shown). The Xf∆*pilJ* strain was placed in storage at -80°C in PD2 with 7% DMSO.

**Figure 3:**
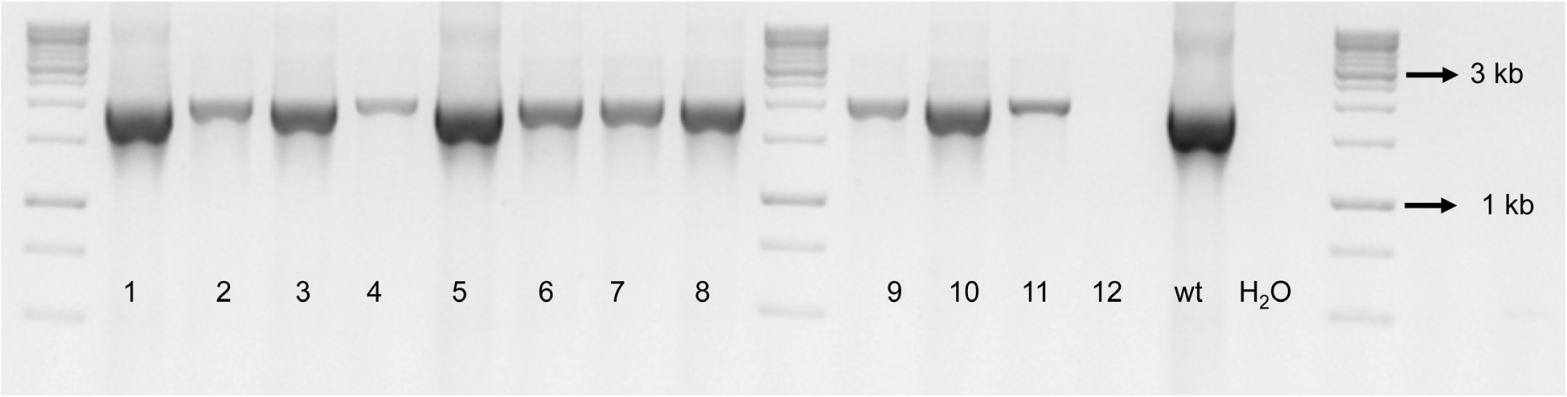
Mixture of wild-type and mutant *Xylella fastidiosa* strains after first isolation. The XfΔ*pilJ* mutant confirmed in Figure 2 was stored at -80°C, streaked onto periwinkle agar plates amended with kanamycin, and the genotype assessed for 12 single colonies. Each number denotes a single colony. The *pilJ*E/*pilJ*F (EF) primers amplified a 2030 bp band for non-transformed *X. fastidiosa* and no band for the XfΔ*pilJ* mutant. Wild-type *X. fastidiosa* DNA (wt) was used as a positive control for the PCR reaction, while primer reaction without template DNA represented by H_2_O, was used as a negative control.

**Figure 4:**
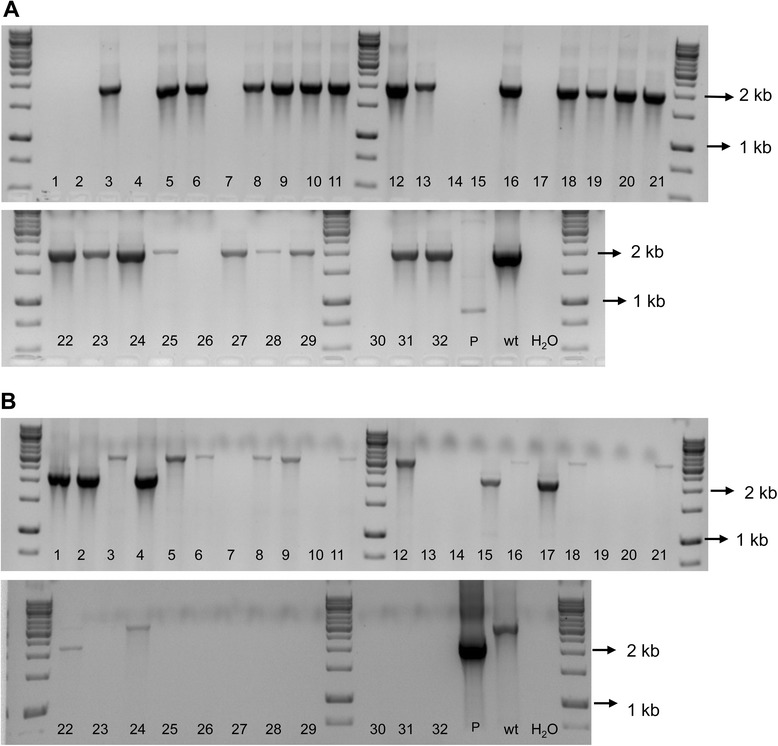
Mixture of wild-type and mutant *Xylella fastidiosa* strains after second isolation. The XfΔ*pilJ* mutant confirmed in Figure 3 (isolate 12) was streaked onto periwinkle agar plates amended with kanamycin and the genotype assessed for 32 single colonies. Each number denotes a single colony. **A**. The *pilJ*E/*pilJ*F (EF) primers amplified a 2030 bp band for non-transformed bacteria and no equivalent bands for the transformed XfΔ*pilJ* mutant strains or the deletion plasmid (P). **B**. The *pilJ*A/*pilJ*D (AD) primers amplified a 3082 bp band for non-transformed cells and a 2200 bp fragment from the XfΔ*pilJ* strain and the deletion plasmid (P). Wild-type *X. fastidiosa* DNA (wt) was used as a positive control for the PCR reactions, while primer reaction without template DNA represented by H_2_O, was used as negative controls for each PCR reaction.

**Figure 5:**
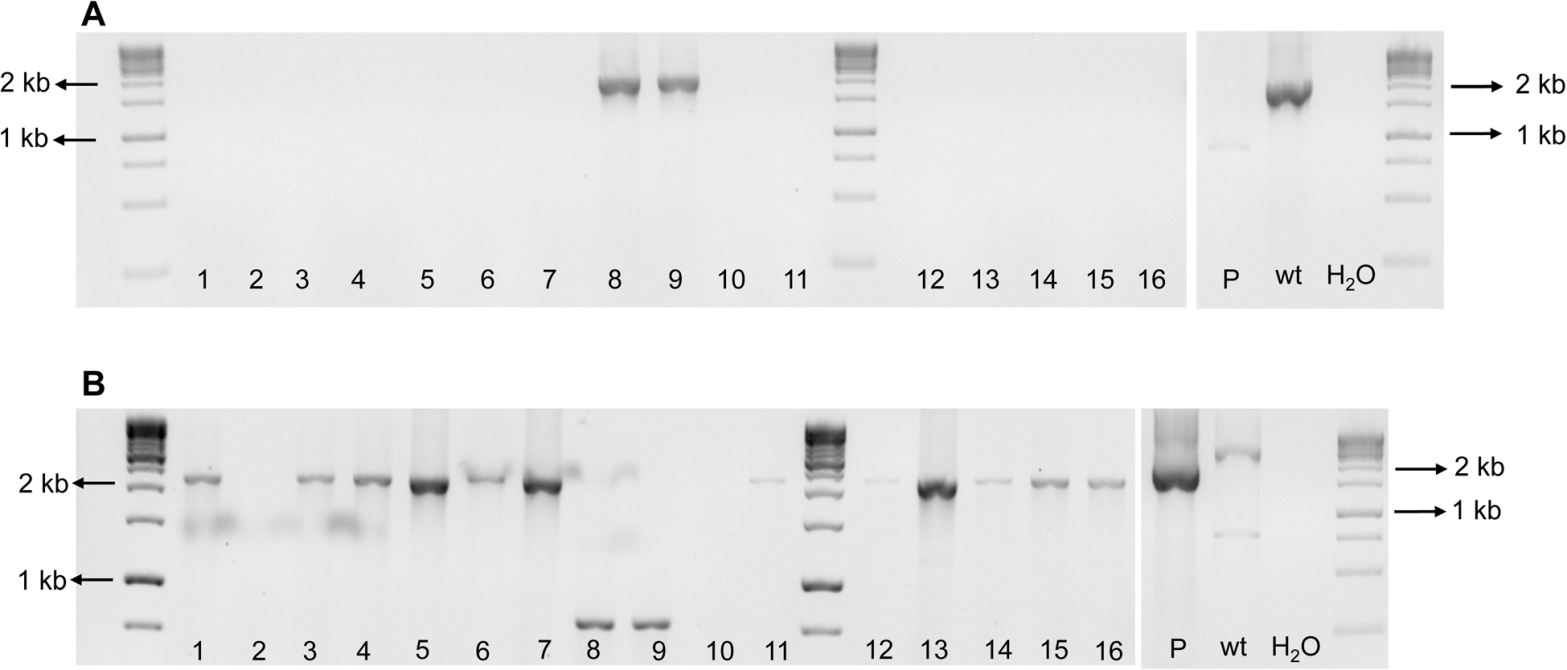
Mixture of wild-type and mutant *Xylella fastidiosa* strains after third isolation. The XfΔ*pilJ* mutants confirmed in Figure 4 (isolates 4 and 17) were streaked onto PW agar plates amended with kanamycin and the genotype assessed for 16 single colonies. Each number denotes a single colony. **A**. The *pilJ*E/*pilJ*F (EF) primers amplified a 2030 bp band for non-transformed bacteria and no equivalent band for the XfΔ*pilJ* strains or the deletion plasmid (P). **B**. The *pilJ*A/*pilJ*D (AD) primers amplified a 3082 bp band for non-transformed cells and a 2200 bp fragment from the XfΔ*pilJ* strains and the deletion plasmid (P). Wild-type *X. fastidiosa* DNA (wt) was used as a positive control for the PCR reactions, while primer reaction without template DNA represented by H_2_O, was used as negative controls for both PCR reactions.

The Xf∆*pilJ* strain was streaked onto PW agar plates amended with kanamycin after -80°C storage. The Xf∆*pilJ* mutant was observed to have behavioral phenotypes different from that previously observed for the mutant before storage but similar to wild-type *X. fastidiosa* (data not shown). The genotype of the Xf∆*pilJ* mutant was therefore assessed by PCR. Xf∆*pilJ* mutant directly from the -80°C stock was streaked onto PW agar plates with kanamycin to obtain single colonies and assessed by PCR. Of the twelve single colonies analyzed with the EF primers, 11 gave bands suggestive of non-transformed cells (Figure 3). Colony 12, which lacked a fragment with the EF primers, suggesting it was the Xf∆*pilJ* mutant strain, was streaked onto PW-kanamycin for a second round of single colony isolation. Subsequently, 32 colonies from round two were transferred to new PW plates with kanamycin and used for PCR with the AD and EF primer sets (Figure 4). Five colonies (colonies 1, 2, 4, 15, 17) appeared to be the Xf∆*pilJ* mutant strain, as they did not give a band with the EF primers and had a 2200 bp band with the AD primers, while 11 colonies exhibited results typical of non-transformed cells, as they gave bands with the EF primers and a 3082 bp band with the AD primers. Mixed colonies of non-transformed and transformed cells were also observed (colony 22); where the EF primers amplified a band indicating the non-transformed strain was present, and the AD primers amplified a mutant size band indicating that the Xf∆*pilJ* mutant was also present. Samples that appeared to be non-transformed or failed to amplify a band with AD primers, whether they amplified a band with EF primers or not, were not further analyzed. Two of the five transformed colonies were streaked onto PW agar plates with kanamycin to obtain single colonies for a third round of isolation. Of the 16 colonies examined in round three, 13 gave the mutant phenotype with the EF and AD primers (Figure 5). Again, those colonies appearing to be non-transformed, failing to amplify a band with AD primers, or giving a very small band with the AD primers were not further examined. Four of the Xf∆*pilJ* mutants were restreaked onto PW with kanamycin and assessed by real-time PCR for the presence of the *pilJ* gene (data not shown). None of these colonies were positive for the *pilJ* gene, therefore the samples were stored at -80°C in PD2 with 7% DMSO. This phenomenon of contamination by non-transformed cells was not limited to the Xf∆*pilJ* mutant strain, but observed with a number of our *X. fastidiosa* deletion mutants (data not shown).

**Figure 6:**
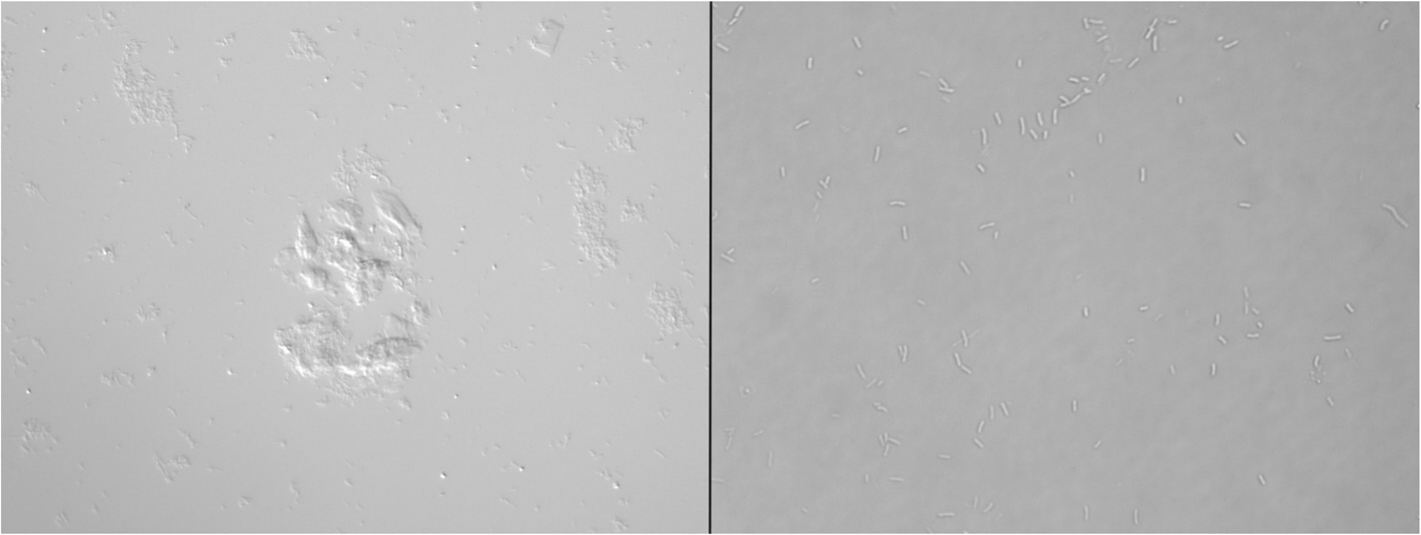
Aggregation of *Xylella fastidiosa* and *Escherichia coli*. A suspension of *X. fastidiosa* (left) or *E. coli* (right) in SCP buffer five minutes after vigorous resuspension by vortexing and pipetting. Twenty microliters was pipetted onto slides and viewed by microscopy at 40X. While *E. coli* cells dispersed, *X. fastidiosa* present as aggregates, and could not be evenly dispersed.

Mixtures of constructed kanamycin-resistant Xf∆*pilJ* mutant and non-transformed strains may have occurred due to spontaneous antibiotic resistant mutation in wild-type cells, high aggregation rate of *X. fastidiosa*, or a combination of both events. The ability of bacteria to develop spontaneous resistance to antibiotics is a well known phenomenon [18]. Sub-optimal antibiotic conditions provide particularly favorable conditions for these mutant strains to emerge. Of note, *X. fastidiosa* mutants were grown at standard, not sub-optimal, kanamycin concentrations [11]. Mixed aggregates of susceptible wild-type with kanamycin-resistant constructed Xf∆*pilJ* mutant strains may also explain the findings. *X. fastidiosa* characteristically and spontaneously forms aggregates that are not easily dispersed, compared to bacteria such as *Escherichia coli*, even after vigorous resuspension by mixing and pipetting (Figure 6). Presumably both aggregated and planktonic cells were transformed during mutant generation, and mixed aggregates of transformed and non-transformed cells formed subsequently. The aggregate formation may result in decreased susceptibility to antibiotics by the non-transformed bacteria [19,20]. After transformation when plating onto selective media (kanamycin) non-transformed bacteria may be “protected” from antibiotics by antibiotic resistant bacteria present in the aggregate, as is found in biofilms [21].

**Figure 7:**
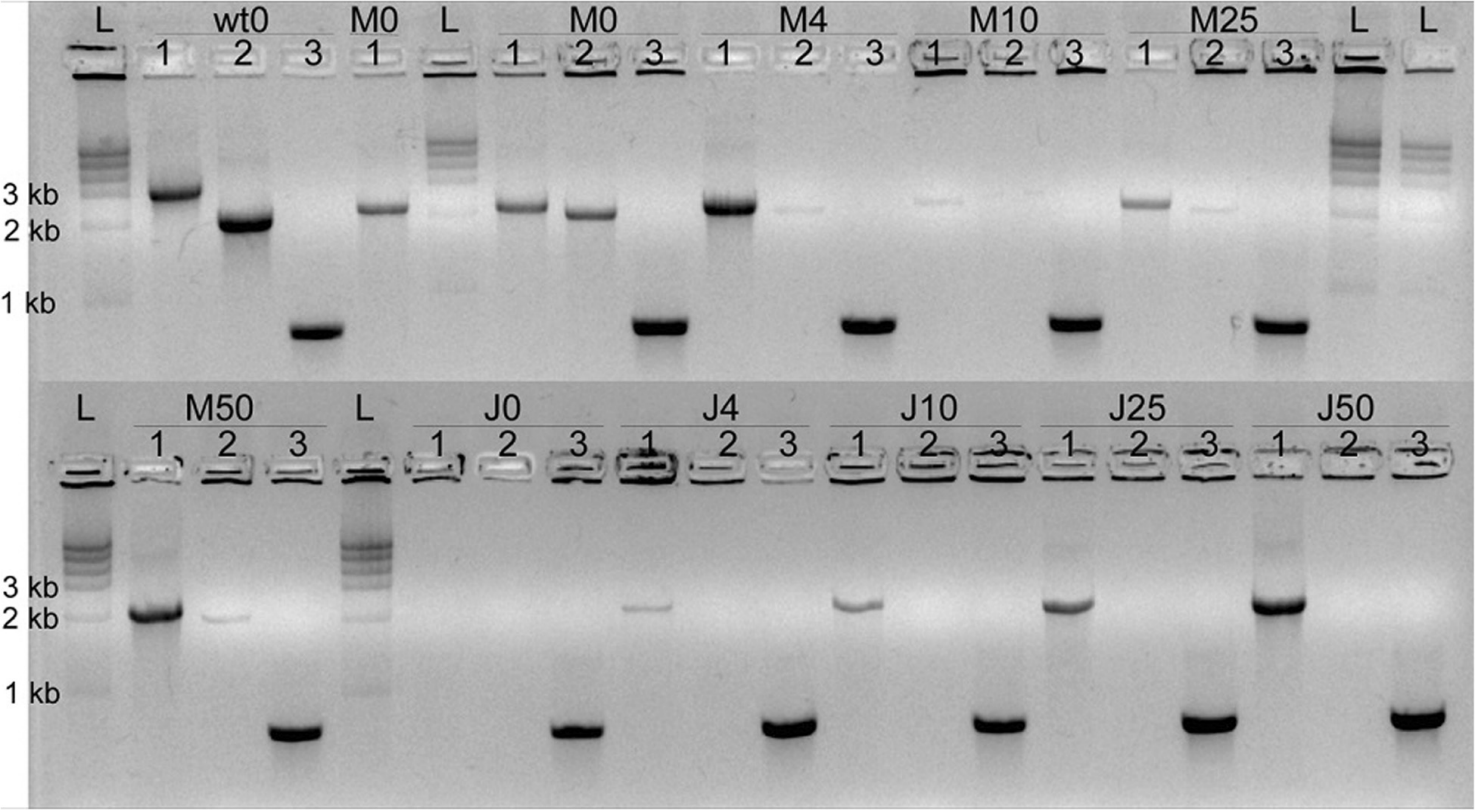
Protection of wild-type *Xylella fastidiosa* from antibiotic selection pressure. Wild-type *X. fastidiosa* (wt), XfΔ*pilJ* mutant (J), or an equal mixture of both (M) were grown in PD2 liquid media before being plated onto agar plates with 0, 4, 10, 25, or 50 μg/mL kanamycin, and tested by PCR. The *pilJ*A/*pilJ*D (AD) primers amplified a 3082 bp band for wild-type cells and a 2200 bp fragment from the XfΔ*pilJ* strain. The *pilJ*E/*pilJ*F (EF) primers amplified a 2030 bp band for wild-type bacteria and no equivalent bands for the XfΔ*pilJ* mutant strains. For mixed samples, the AD primers amplified a Xf *pilJ* strain fragment and the EF primers amplified a wild-type band. The RST31/33 (RST) primers confirmed that the bacteria were *X. fastidiosa*. Wells of each cell type and kanamycin concentration condition are numbered as follows: (1) AD amplification, (2) EF amplification, and (3) RST amplification.

We tested the importance of mutants protecting wild-type bacteria from antibiotics by growing cells (wild-type *X. fastidiosa*, Xf∆*pilJ* mutant strain, or an equal mixture) for 24 hours and plating them on PW with various concentrations of kanamycin, as is done for transformations. The wild-type-only *X. fastidiosa* sample gave the expected bands with all the primers tested when grown on 0 μg/mL of kanamycin (Figure 7). At higher concentrations of antibiotics no bacterial growth was observed on agar plates, therefore PCR could not be conducted. The Xf∆*pilJ*-only mutant sample grew on all concentrations of antibiotics tested and gave the expected fragments with the AD and RST primers, while the EF primers did not produce a fragment, as expected. In one of three trials, PCR analysis of the plated mixed colonies of wild-type and the Xf∆*pilJ* mutant showed the presence of both strains; AD primers amplified the Xf∆*pilJ* mutant 2200 bp band, and the EF primers amplified a wild-type 2030 bp band at all concentrations of antibiotics tested except 10 μg/mL. These results indicate the presence of a small number of wild-type bacteria even on PW agar plates containing 50 μg/mL of kanamycin. While spontaneous antibiotic resistance cannot be ruled out, our results suggest that protection of the wild-type strain in mixed cell populations readily occurs, particularly as no wild-type-only samples grew on any kanamycin concentrations. In addition, the fact that the wild-type EF band was faint and not observed on one kanamycin PW plate (10 μg/mL concentration) may indicate that in our original findings (Figure 2) the number of wild-type cells in the mixed population may have been below detection level by conventional PCR.

Overall, it is possible that following transformation, colonies on selection plates may not have arisen from a single cell but from an aggregate containing a mix of the transformed and non-transformed cells. The presumed Xf∆*pilJ* mutant clone was then stored in PW amended with 7% DMSO. After thawing and refreezing the non-transformed bacteria present may grow and attain populations that can affect the overall population dynamics and skew results of phenotypic assays as observed after recovery of stored Xf∆*pilJ*, suggesting they were more fit for the freeze/thaw process.

Various aspects of *X. fastidiosa* aggregation have been reported. *X. fastidiosa* was found to aggregate into star-like clusters in microfluidic chambers under conditions of xylem flow [5]. The extent of aggregation is known to be dependent on media [22], xylem fluid source [23], and grapevine xylem chemistry [24]. Calcium can increase the ability of the bacterium to form aggregates while calcium chelators, such as ethylene glycol tetraacetic acid (EGTA) and 1,2-bis(*o*-aminophenoxy)ethane-*N*,*N*,*N*′,*N*′-tetraacetic acid acetoxymethyl ester (BAPTA/AM), cause a decrease in aggregation [25]. It would be interesting to determine if growth in media that promotes planktonic suspensions, followed by transformation, would reduce the possibility of mixed clones when generating *X. fastidiosa* mutants.

## Conclusions

While it is possible to make targeted deletions in *X. fastidiosa,* complications may arise due to spontaneous antibiotic resistance and/or cell aggregates formed by the bacteria. The presence of aggregates may allow non-transformed bacteria to survive on PW kanamycin agar plates. At least three passages of single colony isolation followed by PCR may be required to minimize the amount of non-transformed cells contaminating the transformants. It may also be prudent to perform single colony isolation after retrieval from storage before use of mutant strains in assays.

## Abbreviations

AD: primers *pilJ*A and *pilJ*D
BAPTA/AM: 1,2-bis(*o*-aminophenoxy)ethane-*N*,*N*,*N*′,*N*′-tetraacetic acid acetoxymethyl ester
bp: basepair
DMSO: dimethyl sulfoxide
EF: primers *pilJ*E and *pilJ*F
EGTA: ethylene glycol tetraacetic acid
HxfB: hemagglutinin *Xylella fastidiosa* B
LB: Luria Bertani
PCR: polymerase chain reaction
PD2: Pierce’s Disease 2
PW: Periwinkle wilk
RT: real time
SCP: Succinate-citrate-phosphate
XadA: *Xanthomonas* adhesin-like protein A
XatA: *Xylella fastidiosa* autotransporter A.
